# Digitizing a Therapeutic: Development of an Augmented Reality Dual-Task Training Platform for Parkinson’s Disease

**DOI:** 10.3390/s22228756

**Published:** 2022-11-12

**Authors:** Jay L. Alberts, Ryan D. Kaya, Kathryn Scelina, Logan Scelina, Eric M. Zimmerman, Benjamin L. Walter, Anson B. Rosenfeldt

**Affiliations:** 1 Department of Biomedical Engineering, Lerner Research Institute, Cleveland Clinic, 9500 Euclid Ave., Cleveland, OH 44195, USA; 2 Center for Neurological Restoration, Neurological Institute, Cleveland Clinic, 9500 Euclid Ave., Cleveland, OH 44195, USA

**Keywords:** Parkinson’s disease (PD), dual-task, augmented reality, gait, postural instability

## Abstract

Augmented reality (AR) may be a useful tool for the delivery of dual-task training. This manuscript details the development of the Dual-task Augmented Reality Treatment (DART) platform for individuals with Parkinson’s disease (PD) and reports initial feasibility, usability, and efficacy of the DART platform in provoking dual-task interference in individuals with PD. The DART platform utilizes the head-mounted Microsoft HoloLens2 AR device to deliver concurrent motor and cognitive tasks. Biomechanical metrics of gait and cognitive responses are automatically computed and provided to the supervising clinician. To assess feasibility, individuals with PD (*N* = 48) completed a bout of single-task and dual-task walking using the DART platform. Usability was assessed by the System Usability Scale (SUS). Dual-task interference was assessed by comparing single-task walking and walking during an obstacle course while performing a cognitive task. Average gait velocity decreased from 1.06 to 0.82 m/s from single- to dual-task conditions. Mean SUS scores were 81.3 (11.3), which placed the DART in the “good” to “excellent” category. To our knowledge, the DART platform is the first to use a head-mounted AR system to deliver a dual-task paradigm and simultaneously provide biomechanical data that characterize cognitive and motor performance. Individuals with PD were able to successfully use the DART platform with satisfaction, and dual-task interference was provoked. The DART platform should be investigated as a platform to treat dual-task declines associated with PD.

## 1. Introduction

Nearly six decades have passed since Ivan Sutherland contemplated a computer interface that blurred the line between the digital and physical worlds [1]. Sutherland proposed creating a see-through head-mounted display in which users could see digital images superimposed over the real world [2]. Eventually, that concept would be termed augmented reality (AR). Augmented reality consists of the following characteristics: (1) it combines the real and virtual worlds, (2) it is interactive in real-time, and (3) digital images are registered in three dimensions (3D) [3]. A recent summary of publication trends in virtual reality (VR), AR, and mixed reality (MR) indicated that manuscripts in the neurosciences (*N* = 1000+) and rehabilitation (*N* = ~750) ranked second and third in terms of the number of peer-reviewed publications using these technologies between 2009 and 2020 [4]. The substantial number of publications in neurosciences and rehabilitation indicate that AR, VR, and MR technologies may, on the surface, be useful in the evaluation and treatment of individuals with a neurological deficit, such as Parkinson’s disease (PD). The importance and need to objectively quantify the effects of PD on upper and lower extremity performance is well documented [5,6,7,8,9], and AR has the potential to meet that need.

The “ultimate” technology platform for the delivery of rehabilitation was recently conceptualized: an immersive system consisting of a wearable device that can provide objective outcomes to automatically scale the difficulty of therapy [10]. Despite the potential of utilizing immersive technology such as AR to evaluate and treat postural instability and gait disturbances (PIGD) in individuals with PD, its integration into clinical practice has been sparse [11]. Speculation underlying the barriers to clinical adoption of immersive technology such as AR and VR are plentiful. Our previous success in integrating technology into routine clinical workflows for participants with neurological disease [12,13,14] and injury [15,16,17] suggests that several key concepts must be solved prior to introducing AR or other immersive technologies into the clinical workflow: (1) the ability of AR systems to accurately characterize important aspects of PD movement; (2) the design of a user interface and user experience (UI\UX) that considers the motor and cognitive deficits of PD to ensure that treatment tasks are understandable and engaging; and (3) the platform must be effective at eliciting motor and cognitive deficits common in PD.

Regarding the first limitation related to understanding the accuracy of AR systems, systematic evaluation of the validity and reliability of head-mounted AR devices to accurately quantify human movement shows promise. Our initial work [18], as well as that of others [19,20,21], sought to evaluate the movement quantification capabilities of the first-generation HoloLens (HL1; Microsoft, Redmond, WA, USA), a head-mounted immersive AR system. Overall, these projects indicate that the positional data from the HL1 is of sufficient quality and quantity to accurately characterize lower extremity performance. Gait velocity, step length, cadence [18,20], locomotion distance [18], and head movement [21] outcomes from the HL1 were comparable to the same outcomes derived from various motion capture systems in healthy individuals. To facilitate clinical adoption of AR, rigorous testing of gait and mobility impairments in individuals with less typical gait patterns and in non-linear movement patterns should be assessed. In PD, gait velocity, step length, and cadence calculated from the HL1 were comparable to a motion capture system [20]. Turning is a complex motor skill that is associated with fall risk in older adults [22] and PD [23]; thus, quantifying fall metrics using a head-mounted AR device to assess turning behavior has been recently evaluated [24]. Data from the second-generation HoloLens2 (HL2), which offers a larger field of view, new generation holographic processing unit for enhanced visual display, eye-tracking capabilities, and improved hand tracking to improve holographic interaction, were compared to 3D motion capture data to evaluate turning. The results indicated that the HL2 provided valid measures of turn duration and turn velocity [24]. The rigorous validation of the first- and second-generation HL in both healthy adults and individuals with PD addresses the first barrier to clinical adoption; medical providers can trust the biomechanical data derived from the HL2, making it a prime candidate for the delivery of an accepted approach to treating PD lower extremity dysfunction.

Immersive AR and VR platforms have the ability to examine information processing and provide insight into the disease process by measuring dual-task interference [25]. While multiple theories on the underlying pathophysiology of dual-task interference in PD exist [26], it is likely that decreased gait automaticity associated with PD results in increased attentional resources being utilized for gait. Under a dual-task scenario such as walking while performing a cognitive task, limited available attentional resources as a result of PD are divided, and deficits in gait and postural control are exacerbated [27]. Dual-task interference is grounded in the performance of two discrete tasks performed concurrently [25]; an AR platform is capable of instructing, delivering, and objectively measuring performance on a motor and cognitive task delivered simultaneously. Provocation, evaluation, and training of dual-task interference is clinically useful as it mimics conditions of daily life, thereby creating an opportunity for therapeutic or pharmacologic intervention.

The Dual-task Augmented Reality Treatment (DART) platform was designed to instruct and deliver simultaneous motor and cognitive tasks via a head-mounted HL2 in an attempt to provoke dual-task interference. Based on a successful one-on-one therapeutic intervention [28], the ultimate goal of the DART platform is to deliver dual-task training (DTT) as a digital therapeutic intervention involving the simultaneous performance of motor and cognitive tasks, with the goal of improving PIGD symptoms and reducing dual-task interference in PD as part of a clinical trial. The DART platform was designed to provide real-time, objective feedback regarding participant performance and summary data to a physical therapist overseeing the intervention to inform personalized progression of DTT. Prior to initiating a clinical trial utilizing the DART platform, it was necessary to confirm its initial feasibility, usability, and efficacy. This manuscript details the development of the DART platform and provides an overview of its initial feasibility, usability, and efficacy for provoking dual-task interference in people with PD.

## 2. Materials and Methods

### 2.1. DART Platform Development and Utilization

The DART platform utilizes the HL2 (Microsoft Corporation, Redmond, WA, USA) and an iPad (Apple, Cupertino, CA, USA). The HL2 delivers the selected dual-task modules via an application developed in Unity (Unity Software, Inc., San Francisco, CA, USA); the Clinician Dashboard on the iPad, programmed in Swift (Apple, Cupertino, CA, USA), provides synchronous and asynchronous participant performance data to the therapist. The DART platform was designed with the intended clinician user being a physical or occupational therapist and the patient end-user being an individual with PD.

**Figure 1:**
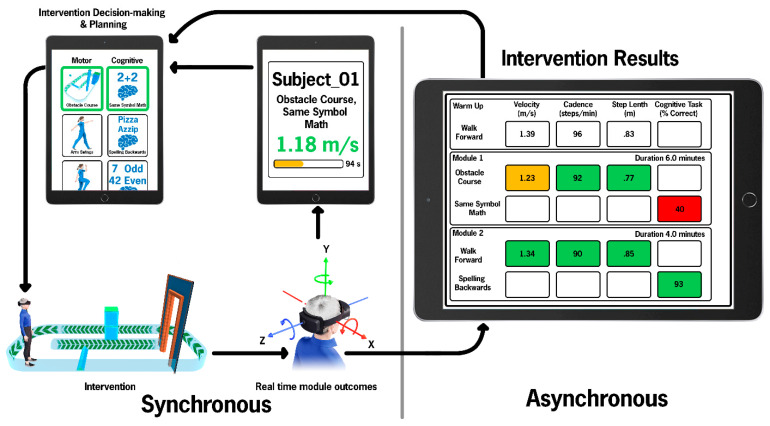
Overview of the synchronous and asynchronous components of the DART Platform. *Intervention Decision-making and Planning:* The therapist selects the motor and cognitive modules on the Clinician Dashboard to create individualized and salient dual-task modules. During the session, the programmed modules are implemented in the therapist-designated order during the intervention. *Intervention:* The patient executes the planned treatment session with the head-mounted HL2, delivered by the digital avatar. *Real Time Module Outcomes:* Data from the HL2 provide instantaneous metrics such as gait velocity and module time progression to the therapist monitoring the session. *Intervention Results:* Comprehensive summary biomechanical gait and turning metrics (not all metrics are provided above) are provided at the end of the session on the Clinician Dashboard.

The DART platform, illustrated in Figure 1, is an interactive platform in which a provider can create patient-specific therapeutic sessions and monitor multiple users simultaneously. The platform consists of a synchronous and asynchronous component.

The synchronous component links the therapist (via the Clinician Dashboard iPad) and the user (via the HL2). The iPad and HL2 devices running the DART software communicate via a SignalR (Microsoft Corporation, Redmond, WA, USA) server hosted on an Amazon Web Services EC2 (Amazon.com, Inc., Seattle, WA, USA) instance with the use of WebSockets. The SignalR server enables persistent wireless communication between devices using the Transmission Control Protocol (TCP) protocol. As designed, a single therapist could technically simultaneously monitor up to ten participants completing DART.

Throughout the session, the HL2 transmits a status update message to the iPad in the form of a JSON file. Communication between the iPad and HL2 occurs every five seconds, providing real-time data to the therapist monitoring the treatment session. This JSON file contains information regarding the current module in progress: the physical and cognitive task being performed, the time elapsed in the module, and the current walking speed of the participant derived from the HL2 headset positional data as it changes over time.

The asynchronous component of the DART platform facilitates the planning and review of the DART sessions. Via the Clinician Dashboard, the therapist can pre-program custom protocols using a variety of modules, as detailed below. During a module, the HL2 application records the headset position as a vector, headset rotation as both Euler angles and quaternions, and eye gaze direction as a vector. Following module performance, the biomechanical data are saved to a Comma Separated Values (CSV) file, and the cognitive data are saved to a JavaScript Object Notation (JSON) file. These files are then transmitted to a HIPAA-compliant Amazon Web Service (AWS) Simple Storage Service (S3) bucket for the calculation of detailed cognitive and motor outcomes.

### 2.2. Creating and Implementing Patient-specific DTT Sessions

The Clinician Dashboard viewable on the iPad enables the therapist to create patient-specific sessions by selecting one of 14 motor tasks and combining them with one of the 17 cognitive tasks. The provider has the ability to customize the length of the session. The variety of motor and cognitive tasks aims to provide digital versions of tasks used in a previous dual-task intervention clinical trial [28].

### 2.3. Motor Task Development

**Figure 2:**
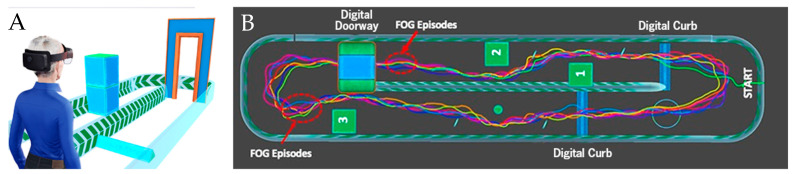
The Obstacle Course of the DART platform. (**A**) User view. The individual is presented with digital pillars, obstacles, curbs, and/or a doorway that must be navigated. To further increase task demands, a secondary cognitive task can be presented simultaneously and delivered by the HL2 DART platform. (**B**) A top-down view of the obstacle course with representative data from a single user. Each colored line represents one lap that the individual completed during a 180 s trial; the numbered boxes represent three pillars that required sidestepping to avoid. Freezing of gait (FOG) episodes, indicated by red circles, occurred prior to entering the doorway and at the end of the turn when presented with a narrowed passage.

The motor tasks were developed to treat hallmark gait characteristics of early to mild-to-moderate stage PD. Neurodegeneration of the basal ganglia results in decreased motor automaticity, making it difficult for individuals with PD to achieve and sustain the rhythmic movement necessary for typical gait patterns [29]. Gait deficits in PD have been well characterized [30], and include decreased gait velocity, decreased step length, increased cadence, decreased arm swing, and freezing of gait. To address the most frequent PD gait impairments, 14 DART motor tasks, provided in Table 1, were created. Notably, customization within a given task is possible, such as setting the step length during the Footprint Targets and manipulating the number and position of obstacles in the Obstacle Course. An overview of the interactive digital Obstacle Course is shown in Figure 2. Examples of configurations for the Obstacle Course include avoiding or stepping over digital obstacles and curbs and passing through a digital doorframe to replicate walking through confined spaces, which is known to trigger freezing of gait in PD [30].

### 2.4. Cognitive Task Development

Cognitive tasks were developed using an evidence-based approach that identified the cognitive domains that result in dual-task declines in PD [31,32,33]. Cognitive performance is typically classified into domains of functioning [34]. For the DART platform, the domains of attention, memory, language, and executive function were selected based on previous training protocols [28]. In sum, 17 tasks were developed within the four cognitive domains. The tasks can be scaled depending on education level and cognitive abilities of the participant. For example, in the spelling backwards task (attention domain), difficulty may be scaled by using words that are three, four, or five+ letters. 

### 2.5. The Digital Avatar Experience

**Figure 3:**
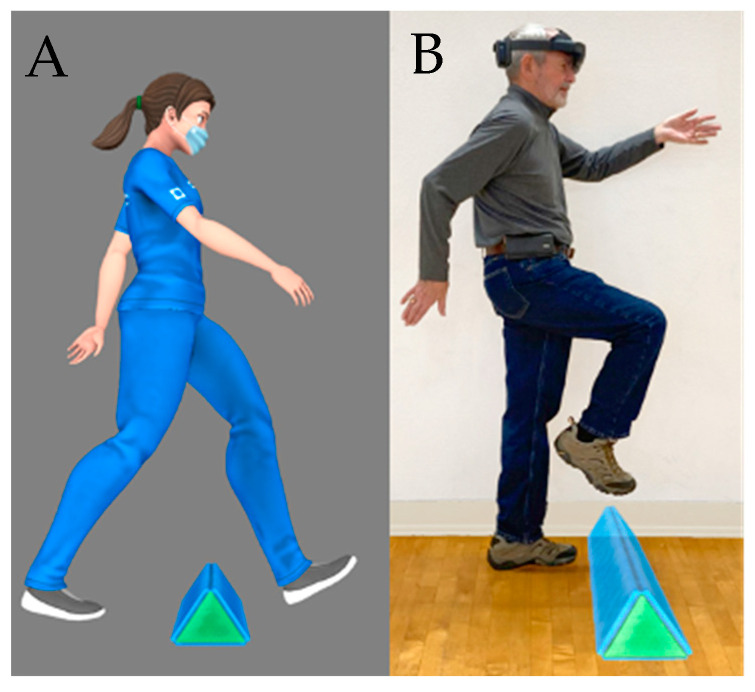
(**A**) Donna, the digital DART avatar, demonstrating stepping over an object during the obstacle course instructions. (**B**). An individual with PD navigating the same object in the obstacle course. The object on the floor is a hologram only visible to the user wearing the HL2.

A digital avatar was created to autonomously guide the patient through the therapy session. Donna, the avatar created in the image of a physical therapist, guides the user through each module by modeling the physical task and providing an auditory explanation of the cognitive task with a corresponding example (Figure 3). The avatar provides periodic cues to engage and ensure the participant is performing the motor task to their full potential (i.e., during forward walking, the avatar provides an auditory reminder to take long steps leading with a heel strike).

### 2.6. Synchronous User and Provider Experience

The participant dons the HL2 headset and depresses a holographic button to launch their programmed DART session. During each module, the gait speed (m/s) and time remaining in the module are communicated from the HL2 to the Clinician Dashboard every five seconds; thus, the therapist is almost instantaneously updated on the user’s status. The clinician has the ability to pause, restart, terminate, or skip any treatment modules during a session based on the instantaneous data or in-person observation.

### 2.7. Asynchronous Clinician Interface: Post-DTT Session Review of Biomechanical and Cognitive Outcomes

Summary biomechanical metrics for each of the modules completed are automatically calculated. Positional data from the IMU of the HL2 are used to calculate biomechanical gait variables using previously validated algorithms [18,24]. Gait variables included: gait velocity (m/s), cadence (steps/min), step length (m), average turn velocity (deg/s), peak turn velocity (deg/s), turn time (s), and number of turns. The algorithms used to calculate biomechanical outcomes are robust, as data are provided even during unconstrained and unpredictable movements across a range of directions and speeds. The provider is able to access summary data via the Clinician Dashboard on the iPad for review and subsequent session planning.

To better understand dual-task interference and aid with intervention progression during the clinical trial, the summary metrics are color-coded based on the percent decline from the single-task performance (Figure 1); any module involving forward walking is compared to the single-task forward walking, etc. Biomechanical outcomes are green if their value is 90+% of the warm-up value, yellow if between 80–89% of the warm-up, and red if <80% of the warm-up. The initial 10% cutoff was modeled after the Timed Up and Go subtask of the Mini-BESTest, where a >10% worsening of performance indicates dual-task impairment [35]. Discrete cognitive tasks are green if the participant responded correctly to 85–100% of the questions, yellow if 70–84% correct, and red if <70% correct. Open-ended questions (i.e., “*Provide directions from your house to the nearest grocery store*”) are not scored. When utilizing the DART platform as an interventional tool, the color-coded objective feedback informs task grading (increasing or decreasing task difficulty).

#### 2.7.1. Study Design

As part of a larger clinical trial (NCT04634331), a cross-sectional study of individuals with PD completed a System Usability Scale (SUS) immediately following a single use of the DART platform. Inclusion criteria for the clinical trial included diagnosis of idiopathic PD, Hoehn and Yahr I-III, ability to ambulate at least 10 minutes without an assistive device, and self-reported gait or balance deficits. Exclusion criteria included surgical procedures for PD, a diagnosis of dementia or neurocognitive deficit that would impair the ability to provide consent, a musculoskeletal or cardiopulmonary condition that would restrict walking activity, other neurological disorder(s), and three or more errors on the Short Portable Mental Status Questionnaire (SPMSQ) [36].

In order to determine whether the DART platform induced dual-task interference, all participants performed a 10- to 15-minute self-directed protocol to familiarize them with the technology and introduce them to the digital avatar. Administration times varied slightly due to the self-paced nature of the protocol (i.e., participants could repeat instructions). A portion of the protocol included a bout of single-task forward walking followed by navigation of a digital obstacle course while performing a language task. For the language task, participants were delivered five words, of which four of the five were related (i.e., run, walk, sleep, jump, and crawl). The participant was asked to verbalize the word that was unrelated to the other four (i.e., sleep). A standard response time of five seconds was used across all participants prior to the start of the next word grouping.

The SUS is widely accepted as a valid measure of technology usability in technology systems and application development to quickly and systematically evaluate technology usability [37]. Scores for the SUS range from 0–100, with higher scores indicating increased perceived usability. Cut-off scores have been established, with a raw SUS score of 68 indicating a 50th percentile rank or a grade of “C” using a curved grading scale [38]. Achievement of an “A” grade requires a composite score of 85 [38] with a corresponding ranking of “excellent” [39].

Clinical symptoms of PD were evaluated by a trained assessor using the Movement Disorder Society—Unified Parkinson’s Disease Rating Scale, motor portion (MDS-UPDRS III) [40]. Assessments were conducted in the on-antiparkinsonian medication state (~1 h post-antiparkinsonian medication).

#### 2.7.2. Statistical Analysis

Summary statistics of the participants’ demographic information were compiled. To determine usability, the SUS scores were summarized. To determine whether usability scores were associated with individual characteristics, Pearson correlations were calculated between SUS scores and select demographic variables (i.e., age, disease duration, disease severity as measured by the MDS-UPDRS III, and years of education). To determine the feasibility of provoking dual-task interference, gait velocity (m/s), cadence (steps/min), step length (m), and peak turn velocity (deg/s) from the DART platform were summarized for single-task forward walking and dual-task obstacle course walking. Differences between single-task forward walking and dual-task obstacle course walking for each outcome metric were assessed using paired *t*-tests.

## 3. Results

A total of 48 participants completed the single DART session. Participant demographics are summarized in Table 2.

### 3.1. Feasibility and Usability

All participants were able to successfully complete the entire DART protocol; no adverse events occurred during its use. Following the single use of the DART platform, the mean SUS score was 81.3 (11.3), placing the DART platform in the “good” to ”excellent” category [39]. There was no significant correlation between the SUS score and select demographic variables, including disease severity as measured by the MDS-UPDRS III (*p* > 0.05).

### 3.2. The DART Platform Induces Dual-Task Interference in Individuals with PD

**Figure 4:**
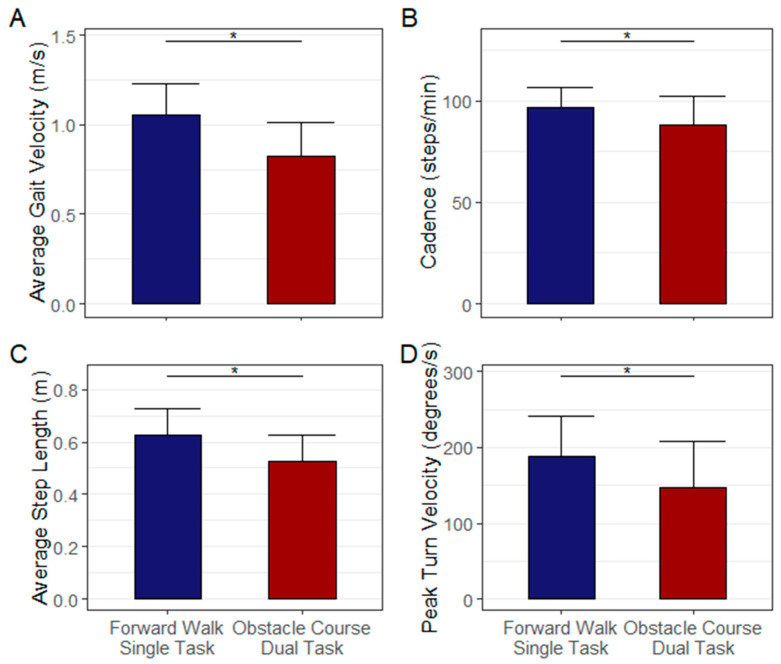
Summary gait and turning metrics derived from the HoloLens2 data for continuous forward walking (blue) and walking through the digital obstacle course under dual-task conditions (red). Gait measures while performing the dual-task obstacle condition resulted in significantly slower average gait velocity (Panel **A**), decreased cadence (Panel **B**), shorter average step length (Panel **C**), and lower peak turning velocity (Panel **D**). These data indicate that the DART protocol is an effective method of eliciting dual-task interference in Parkinson’s disease. The * denotes a significant difference from single-task to dual-task performance (*p* < 0.05).

Gait data from one participant could not be included in the gait analyses due to a technology error. For the remaining 47 participants, the DART platform was able to detect differences between single-task forward walking and dual-task obstacle course performance. The group experienced a general decline in dual-task performance with the addition of a complex motor-cognitive task. Participants exhibited decreased average gait velocity (1.06 (0.17) to 0.82 (0.19) m/s, *p* < 0.001), cadence (96.9 (9.6) to 88.2 (14.1) steps/min, *p* < 0.001), average step length (0.63 (0.10) to 0.53 (0.10) m, *p* < 0.001) and peak turning velocity (187.9 (52.8) to 146.9 (61.3) deg/s, *p* < 0.001) from single- and dual-task obstacle course performance, respectively (Figure 4).

## 4. Discussion

The DART platform leverages the potential of a head-mounted AR device to induce and quantify dual-task interference in individuals with PD. The visual, auditory, and data mining capabilities of the platform were utilized to deliver clear instructions prior to task performance, create engaging and focused dual-task paradigms, and provide objective feedback following module completion. To our knowledge, the DART platform is the first to use a head-mounted AR system to deliver an intervention that provokes dual-task interference while simultaneously providing near real-time biomechanical data characterizing patient performance to the clinician.

Differences in gait and turning variables, from continuous walking and turning to navigating the obstacle course under dual-task conditions support the concept that an AR environment delivered via a head-mounted device such as the HL2 can provoke dual-task interference. The marked decline in spatiotemporal gait and turning variables during the dual-task obstacle performance compared to forward walking indicates that the participants were engaged with the requirements of each task and locomotion was adjusted. Notably, the avatar demonstrated movements in a manner that were very humanlike, as they were fluid and the joint patterns were modeled after human actions. The next steps in terms of refining the avatar are to create additional options for participants to select an avatar that is most engaging to them (e.g., expand racial options, sex, etc.).

Complementary studies support the feasibility of using head-mounted AR devices in PD. Individuals with PD typically experience no motion sickness, find the device relatively comfortable on their head and neck, and are able to clearly see the holograms and hear the auditory stimuli [41]. A reasonable barrier to the utilization of AR in the treatment of PD is a fear that older adults may not be able to use the technology or may not find the technology engaging. Initial usability data from this project indicate people with PD are able to use a well-conceived AR therapeutic and rate the DART platform favorably based on a SUS of 80+points, which equates to a “good” to “excellent” rating [39]. Using more recent methods of scoring and interpreting the SUS, the DART platform received an “A” grade on the curved grading scale, corresponding to the 90–95th percentile of scores, which is above the accepted industry standard of 80 or above [38]. Widely used websites and applications such as Amazon and Gmail scored 81.8 and 83.5, respectively [38]. A report by the Pew Research Center reported that only 26% respondents aged 65+ felt “very confident when using computers, smartphones, or other electronics” as compared to 60% of respondents in the ages 30–49 years old [42]. It is important to note that most consumer-based computer and smartphone technology utilizes UI\UX approaches that do not contemplate the potential cognitive and/or motor declines that older adults experience, and certainly fail to consider PD-related changes in neurological function in the design process. Considering that the mean age of the participants in this project was 69 years and there was no correlation between SUS scores and demographic variables (including age, disease severity, and disease duration), the high usability scores of the DART platform reinforce the concept that assistive or rehabilitation technology must be developed for the end user in mind, and that it is imperative to consider their cognitive and motor abilities for interactions across the entire application. Overall, the positive usability scores indicate that well-designed AR technology is well-received by people with PD; previous concerns related to usability may reflect a dogmatic view of the ability of older adults and people with PD to effectively use technology.

The DART platform delivers comprehensive motor and cognitive tasks on a lightweight, relatively inexpensive (USD 3500), and commercially available head-mounted AR system that can be safely used without compromising balance or inducing cognitive or physical fatigue [43]. The affordability of the platform and the developed therapeutic content make the DART platform conducive to increasing patient access to a proven therapy in order to counteract the effects of PD on gait. The target population of the DART platform is PD; however, the DART platform has the technical framework necessary to translate dual-task training to other neurologic populations that exhibit deficits, such as individuals with mild cognitive impairment and dementia [44,45], multiple sclerosis [46], and cerebral-vascular accident [47].

In PD, dual-task training improves dual-task performance [28,48,49,50]. Data from this project provides justification for moving forward with our ongoing clinical trial evaluating the DART platform as a digital therapeutic for treating gait declines associated with dual-task interference. The digitization of a proven rehabilitation protocol known to improve upper and lower extremity aspects of gait [28] and the development of an AR application to deliver that digital therapy addresses a fundamental gap in the rehabilitation of PD locomotor declines.

## 5. Conclusions

Individuals with PD were able to effectively and safely use a head-mounted AR device that provoked dual-task declines. The DART platform provides objective and quantitative outcomes related to gait across a continuum of task difficulty, from simple continuous walking to conditions in which participants are avoiding digital obstacles. The demonstrated acceptance of the DART platform in a sample representative of a typical PD population provides support for using AR to deliver a dual-task therapeutic intervention to treat gait and postural deficits in individuals with PD. Pending the results of the on-going clinical trial, utilizing the DART platform to deliver in-home therapy to those in rural and underserved areas will be considered.

## Figures and Tables

**Table 1:** DART motor tasks.

PD Gait Deficit	Task(s)	Task Description
Decreased Gait Automaticity	Forward, Backward, Lateral Walking	Multi-directional walking; auditory cues are provided to address PD gait deficits such as decreased arm swing and heel strike.
Decreased Gait Velocity	Pace Setter	Based on the individual, a gait speed that is 0.2–0.3 m/s greater than their self-selected comfortable speed is programmed as a target. A colored icon provides instantaneous feedback to the participant (red if stopped; orange if ambulating below target velocity; green if meeting or exceeding target velocity).
Decreased Amplitude of Movement	Marching, Heel Kicking, Arm Swings, Targeted Arm Swings	Motor tasks focused on maximizing upper and lower extremity movement amplitude, coordination, and velocity. Targeted arm swings provides the participant with visual targets customized to their arm length in order to increase arm swing path length.
Decreased Step Length	Footprint Targets	Left and right footprint targets are displayed on the floor during forward walking. Step length and width are customizable to each participant.
Impaired Turning, Gait Initiation, and Freezing of Gait	Gait Initiation, Figure of Eight	Gait Initiation: The participant walks to a randomly generated target that is populated in the room. The participant is required to visually scan, turn, and initiate gait toward the next target. Upon arrival at the target, a new target is populated in a randomized location. Figure of Eight: The participant completes repetitive left and right hand turns in the looping figure-of-eight course.
Impaired Object Navigation	Obstacle Course	A closed-loop course with randomly generated obstacles including pillars, large and small obstacles and curbs, and a doorframe. The number and type of obstacles are customizable.
Postural Control Disturbances	Upper and Lower Extremity Targeted Movements, Flying Targets	Targeted Movements: Starting from a static standing position, multi-directional stepping (lower extremity) and reaching (upper extremity) targets appear to challenge anticipatory weight shifting and step initiation. Flying Targets: During gait, upper extremity targets appear and the user must reach for the target while maintaining dynamic postural control during gait.

**Table 2:** Participant demographics and baseline characteristics.

	*N* = 48
Age (years)	69.1 ± 6.2
Gender	
Male	36 (75.0%)
Female	12 (25.0%)
Race	
White	42 (87.5%)
Black	5 (10.4%)
Asian	1 (2.1%)
Ethnicity	
Hispanic or Latino	0 (0%)
Not Hispanic or Latino	48 (100%)
Years of education	16.7 ± 2.3
Employment status	
Employed full-time	4 (8.3%)
Employed part-time	3 (6.3%)
Retired due to PD	12 (25.0%)
Retired by choice	29 (60.4%)
Hoehn and Yahr stage	
I	0 (0.0%)
II	34 (70.8%)
III	14 (29.2%)
Disease duration (years)	6.3 ± 4.2
MDS-UPDRS III score (on medication)	35.7 ± 12.7
Levodopa equivalent dose (mg)	643 ± 320
SPMSQ number of errors	0.52 ± 0.58

MDS-UPDRS III = Movement Disorders Society Unified Parkinson’s Disease Rating Scale Motor Exam; SPMSQ = Short Portable Mental Status Questionnaire. Summary statistics presented as mean ± standard deviation or N (%) for categorical data.

## Data Availability

The data presented in this study are available on request from the corresponding author.
